# Indispensable role of mitochondria in maintaining the therapeutic potential of curcumin in acute kidney injury

**DOI:** 10.1111/jcmm.16934

**Published:** 2021-09-16

**Authors:** Ling Li, Shuyun Liu, Yijie Zhou, Meng Zhao, Yizhuo Wang, Chengshi Wang, Peng Lou, Rongshuang Huang, Liang Ma, Yanrong Lu, Ping Fu, Jingping Liu

**Affiliations:** ^1^ Key Laboratory of Transplant Engineering and Immunology National Clinical Research Center for Geriatrics Frontiers Science Center for Disease‐related Molecular Network West China Hospital of Sichuan University Chengdu China; ^2^ Division of Nephrology and National Clinical Research Center for Geriatrics Kidney Research Institute West China Hospital of Sichuan University Chengdu China

**Keywords:** acute kidney injury, antioxidant, curcumin, inflammation, mitochondria, ROS

## Abstract

Acute kidney injury (AKI) is a serious disease for which effective therapeutic agents are required. The capacity of curcumin (CUR) to resolve renal inflammation/oxidative stress and mitochondrial damage has been reported, but crosstalk between these effects and the consequence of this crosstalk remain elusive. In this study, a hypoxia/reoxygenation (H/R)‐induced renal tubular epithelial cell (TEC) injury model and an ischaemia/reperfusion (I/R)‐induced mouse AKI model were treated with CUR with or without mitochondrial inhibitors (rotenone and FCCP) or siRNA targeting mitochondrial transcription factor A (TFAM). Changes in mitochondrial function, inflammation, the antioxidant system and related pathways were analysed. In vitro, CUR suppressed NFκB activation and cytokine production and induced NRF2/HO‐1 signalling in TECs under H/R conditions. CUR treatment also reduced mitochondrial ROS (mtROS) and mitochondrial fragmentation and enhanced mitochondrial biogenesis, TCA cycle activity and ATP synthesis in damaged TECs. However, the anti‐inflammatory and antioxidant effects of CUR in damaged TECs were markedly abolished upon mitochondrial disruption. In vivo, CUR treatment improved renal function and antioxidant protein (NRF2 and SOD2) expression and reduced oxidative stress (8‐OHdG), tubular apoptosis/death, cytokine release/macrophage infiltration and mitochondrial damage in the kidneys of AKI mice. In vitro, the anti‐inflammatory and antioxidant effects of CUR in damaged kidneys were impaired when mitochondrial function was disrupted. These results suggest mitochondrial damage is a driving factor of renal inflammation and redox imbalance. The therapeutic capacity of CUR in kidneys with AKI is primarily dependent on mitochondrial mechanisms; thus, CUR is a potential therapy for various diseases characterized by mitochondrial damage.

## 1 INTRODUCTION

Acute kidney injury (AKI) is a clinical syndrome characterized by a precipitous decline in renal function due to various causes, such as ischaemia/reperfusion (I/R), sepsis, dehydration and toxins, among which I/R is a leading cause of AKI.^1^ The prevalence of AKI in critically ill patients with sepsis is greater than 40% at admission to the intensive care unit.^2^ The morbidity and mortality associated with AKI remain unacceptably high.^3^ Moreover, the incidence of chronic kidney disease (CKD) is nearly threefold higher among patients with AKI than among those without AKI.^4^ The mechanism of AKI is complicated and involves oxidative stress, inflammation and mitochondrial damage, among other factors.^5^, ^6^ Despite supportive care, including renal replacement therapy, 5‐year mortality after AKI remains high.^7^ Therefore, effective agents to attenuate AKI are still needed.

Polyphenols are an important and broad class of phytochemicals with numerous health‐promoting effects. Curcumin (CUR), a bright yellow polyphenolic constituent of the rhizomes of turmeric and one of the most important polyphenols,^8^, ^9^ has been widely used in folk medicine because of its multiple biological activities, such as its antioxidant, anti‐inflammatory and anticancer properties.^10^ Interestingly, CUR has displayed certain renoprotective effects in animal models of acute and chronic diseases.^11^, ^12^, ^13^ The anti‐inflammatory/antioxidant properties of CUR were proposed as the primary factors responsible for its therapeutic effect in renal diseases.^14^ For instance, CUR intervention decreased activation of the NFκB pathway and the release of cytokines (e.g., TNF‐α, IL‐6, and IL‐1β) and increased the expression of antioxidant proteins (e.g., NRF2 and SOD) in the damaged kidney.^13^ On the other hand, while mitochondrial dysfunction (e.g., elevated mitochondrial reactive oxygen species (mtROS), mitochondrial DNA (mtDNA) and mitochondrial biogenesis defects and energy depletion) is a hallmark of AKI,^6^ CUR has shown the potential to reduce mitochondrial damage after AKI. For example, CUR treatment reduced mitochondrial oxidative stress and improved mitochondrial fission and bioenergetics in rodent models of AKI.^15^, ^16^ Although the anti‐inflammatory/antioxidant effects and mitochondrial protective role of CUR in kidneys with AKI have been reported, the relationship between them and consequence of this relationship remain elusive.

In this study, we aimed to evaluate the renoprotective effect of CUR in cellular and mouse models of AKI and determine whether this effect is primarily dependent on a mitochondrial mechanism. The effective mechanism of CUR in AKI, especially crosstalk between mitochondria and the anti‐inflammatory/antioxidant pathways, was also investigated.

## 2 MATERIALS AND METHODS

### 2.1 Cell culture and treatment

A human renal proximal tubular epithelial cell (TEC) line (HK2) was cultured in Dulbecco's modified Eagle's medium/Ham's F12 medium (DMEM/F12, Invitrogen) supplemented with 10% foetal bovine serum (Gibco, Life Technologies), 50 U/ml penicillin and 50 µg/ml streptomycin in a humidified atmosphere at 37°C with 5% CO_2_. To induce hypoxia/reoxygenation (H/R) injury, HK2 cells were incubated in a hypoxic chamber (≤1% O_2_, 5% CO_2_, 94% N_2_) at 37°C for 24 h and subjected to subsequent reoxygenation (21% O_2_, 5% CO_2_ and 74% N_2_) for 2 h. CUR (5 μM, MedChemExpress (MCE)) was dissolved in DMSO and then diluted with culture medium for cell experiments. To disrupt mitochondrial function, HK2 cells were treated with FCCP (2 μM, MCE) or rotenone (ROT, 2 μM, MCE). To suppress mitochondrial biogenesis, HK2 cells were transfected with mitochondrial transcription factor A (TFAM) siRNA (sense: GAGGGAACUUCCUGAUUCATT; antisense: UGAAUCAGGAAGUUCCCUCTT, 50 nM) and the negative control siRNA (sene: UUCUCCGAACGUGUCACGUTT; antisense: ACGUGACACGUUCGGAGAATT, 50 nM). The siRNA was purchased from GenePharma Biotechnology. HK2 cells were transfected with siRNA using Lipo6000 (Beyotime Biotechnology) according to the manufacturer's instructions, after which the medium was exchanged for fresh complete medium and the cells were cultured for an additional 24 h before experiments.

### 2.2 Cell viability assay

Cells were seeded in a 96‐well plate, incubated under H/R conditions and treated with CUR. After treatment, CCK‐8 solution (Dojindo) was added and incubated with the cells at 37°C for 2 h. The absorbance at 450 nm was measured with a microplate reader (BioTek Instruments Inc.). Cell viability was calculated by normalizing the optical density of the experimental group to that of the control group.

### 2.3 Immunofluorescence (IF) staining

Cells were fixed with 4% paraformaldehyde in PBS for 10 min at room temperature, washed with PBS and permeabilized with 0.3% Triton X‐100 for 10 min. After blocking in 1% BSA for 60 min, the cells were incubated with anti‐Nrf2 (Proteintech) and anti‐NFκB (Proteintech) antibodies overnight at 4°C. After washing with PBS, the cells were incubated with FITC‐ or TRITC‐conjugated secondary antibody (1:500, Abcam) for 1 h at 37°C. Nuclei were visualized by staining with DAPI (1:1000, Sigma). Digital images were captured by fluorescence microscopy (Imager Z2, Zeiss). The mean fluorescence intensity of the images was semiquantitatively analysed with NIH ImageJ software.

### 2.4 ^1^H NMR‐based cell metabolomics assay

Intracellular metabolites were extracted using the chloroform/methanol method as previously described.^17^ The lyophilized aqueous extracts of cells were dissolved in 500 μl of PBS to which 50 μl of TSP (1%, in D_2_O) had been added. ^1^H NMR spectra were measured on a Bruker Avance II 600‐MHz spectrometer (Bruker BioSpin) at 298 K with a 5‐mm PATXI probe. ^1^H NMR spectra were acquired by a NOESY presaturation (NOESYPR1D) pulse sequence (RD‐90°‐*t_1_*‐90°‐*t_m_*‐90°‐acq). The 90° pulse length was adjusted to approximately 10 μs, and 64 scans containing 32,000 data points with a spectral width of 11 ppm were collected. The free induction decays (FIDs) were weighted by an exponential function with a 0.3‐Hz line‐broadening factor and zero‐filled to 32,000 data points prior to Fourier transform (FT). The chemical shifts were referenced to TSP at 0 ppm. The relative concentrations of metabolites were calculated by normalizing the individual peak to the total intensity in each spectrum. A heatmap was prepared, and pathway enrichment analysis of the metabolites was performed using online software (www.metaboanalyst.ca/)]^18^.

### 2.5 Mitochondrial morphology assay

MitoTracker Red (Invitrogen) was used to label mitochondria as previously described.^19^ Briefly, cells were stained with 100 nM MitoTracker Red at 37°C for 30 min. After washing with PBS, the labelled cells were observed by confocal microscopy (Nikon A1, Nikon Corporation).

### 2.6 Measurement of mtROS

The intracellular ROS level was measured by flow cytometry. In brief, cells were incubated with MitoSOX (2 µM, Thermo Fisher Scientific) for 30 min, washed twice with PBS and analysed by flow cytometry (Beckman).

### 2.7 Measurement of ATP concentration

The ATP level in cells or tissues was determined with an ATP Bioluminescence Assay Kit (Beyotime Biotechnology) according to the manufacturer's protocol. Briefly, cells or tissues were lysed in lysis buffer, followed by centrifugation at 12,000 *g* for 5 min at 4°C. The level of ATP was determined by mixing 20 μl of the supernatant with 100 μl of luciferase reagent. The luminescence was then measured with a microplate luminometer (Synergy Mx, Bio‐Tek).

### 2.8 Quantitative real‐time PCR (qPCR)

Total RNA was extracted with TRIzol (Gibco) and reverse‐transcribed into cDNA by an iScript cDNA Synthesis Kit (Bio‐Rad). Real‐time polymerase chain reaction (real‐time PCR) was performed on a CFX96 real‐time PCR detection system (Bio‐Rad) with SYBR Green (Bio‐Rad). The primer sequences are listed in Table S1. qPCR data were analysed with Bio‐Rad CFX Manager software, and relative changes in mRNA expression were calculated by the delta‐delta Ct method with β‐actin as a reference gene.

### 2.9 Western blotting

Cells or tissue samples were lysed in radioimmunoprecipitation assay (RIPA) buffer supplemented with protease and phosphatase inhibitors (Calbiochem). The protein concentration was determined with a BCA Assay Kit (CWBIO). Equal amounts of protein were subjected to 12% SDS‐PAGE and then transferred to PVDF membranes (Merck Millipore). The PVDF membranes were blocked with 5% nonfat milk and incubated with the following primary antibodies overnight at 4°C: anti‐IL‐1β (Abcam), anti‐ICAM‐1 (Proteintech), anti‐HO‐1 (ABclonal), anti‐NrF2 (Abcam), anti‐p‐NFκB (Cell Signaling Technology (CST)), anti‐NFκB (CST), anti‐TFAM (ABclonal), anti‐TOM20 (Abcam), anti‐acetylated‐SOD2 (Ac‐SOD2, Abcam) and anti‐SOD2 (Proteintech). After washing with PBST, the PVDF membranes were incubated with HRP‐conjugated secondary antibody (1:2000, ABclonal, USA) at 37°C for 1 h. Protein bands were detected with a chemiluminescence kit (Merck Millipore) and quantified by ImageJ software (NIH).

### 2.10 Mouse AKI model and treatment

All animal experiments were approved by the Animal Care and Use Committee of West China Hospital, Sichuan University (No. 20211153A) and conducted according to the National Institutes of Health Guide for the Care and Use of Laboratory Animals. Male C57BL/6 mice (25–30 g) were purchased from Chengdu Dossy Experimental Animals Co. Ltd. The animals were housed under standardized conditions with controlled temperature and humidity and a 12‐h light/dark cycle and fed standard chow and tap water *ad libitum*. The mice were randomly divided into four groups (6 per group): the control (Con), I/R, I/R + CUR, and I/R + CUR + ROT groups. The mice were anaesthetized by ip injection of a 1% pentobarbital sodium solution, and the renal artery and vein of the bilateral kidney were occluded for 30 min with a vascular clamp as previously described.^20^ For treatment, CUR (50 mg/kg, dissolved in DMSO/0.9% normal saline, v/v = 2:1) was injected into the peritoneal cavity of the mouse, and mice in the I/R group received vehicle (DMSO in 0.9% normal saline). To disrupt renal mitochondrial function, ROT (5 mg/kg, dissolved in DMSO/0.9% NS, v/v = 2:3) was injected into the kidneys of the mice with an insulin syringe. The body temperature of each animal was maintained at 37°C during surgery. On day 2 after treatment, the mice were sacrificed by an overdose of 1% pentobarbital sodium, and the sera and kidneys were collected for further analysis.

### 2.11 Clinical biochemistry assay

Biochemical analysis of the clinical parameters serum creatinine (CREA), blood urea nitrogen (BUN), alanine aminotransferase (ALT) and aspartate aminotransferase (AST) in the mice was performed on an automatic biochemical analyzer (COBAS INTEGRA 400 plus, Roche) with the appropriate kits.

### 2.12 Transmission electron microscopy (TEM)

Fresh renal tissues from the mice were fixed in a 2.5% glutaraldehyde solution, dehydrated and embedded in Epon resin. Ultrathin sections of the embedded tissues were stained with a 5% uranyl acetate and lead citrate solution and observed by TEM (JEM‐1400Plus, JEOL Ltd.).

### 2.13 Renal histological examination

The kidneys were fixed in 10% formalin and embedded in paraffin, and 5‐μm sections of the embedded kidneys were stained with haematoxylin and eosin (H&E). Cell apoptosis in the frozen kidney sections was measured by TUNEL staining (Promega) according to the manufacturer's protocol. For immunohistochemical (IHC) staining, renal sections were incubated with the following primary antibodies overnight at 4°C: anti‐NGAL (Abcam), anti‐COX IV (Abcam), anti‐HO‐1 (Abcam), anti‐8‐hydroxydeoxyguanosine (8‐OHdG, Abcam), anti‐CD68 (Santa Cruz, CA, USA), anti‐IL‐6 (Proteintech) and anti‐ICAM‐1 (Abcam). The sections were then incubated with HRP‐conjugated secondary antibodies (Millipore) and DAB substrate. Micrographs of the stained sections were captured by light microscopy (Zeiss Imager A2), and the data were quantified with ImageJ.

### 2.14 Statistical analyses

All data are presented as the mean ± SD and were analysed by using SPSS software (version 11.5, IBM Corporation) with one‐way ANOVA or Student's *t*‐test, with *p* < 0.05 used to indicate a significant difference.

## 3 RESULTS

### 3.1 CUR improved cell viability and reduced cytokine secretion in HK2 cells under H/R conditions

**FIGURE 1:**
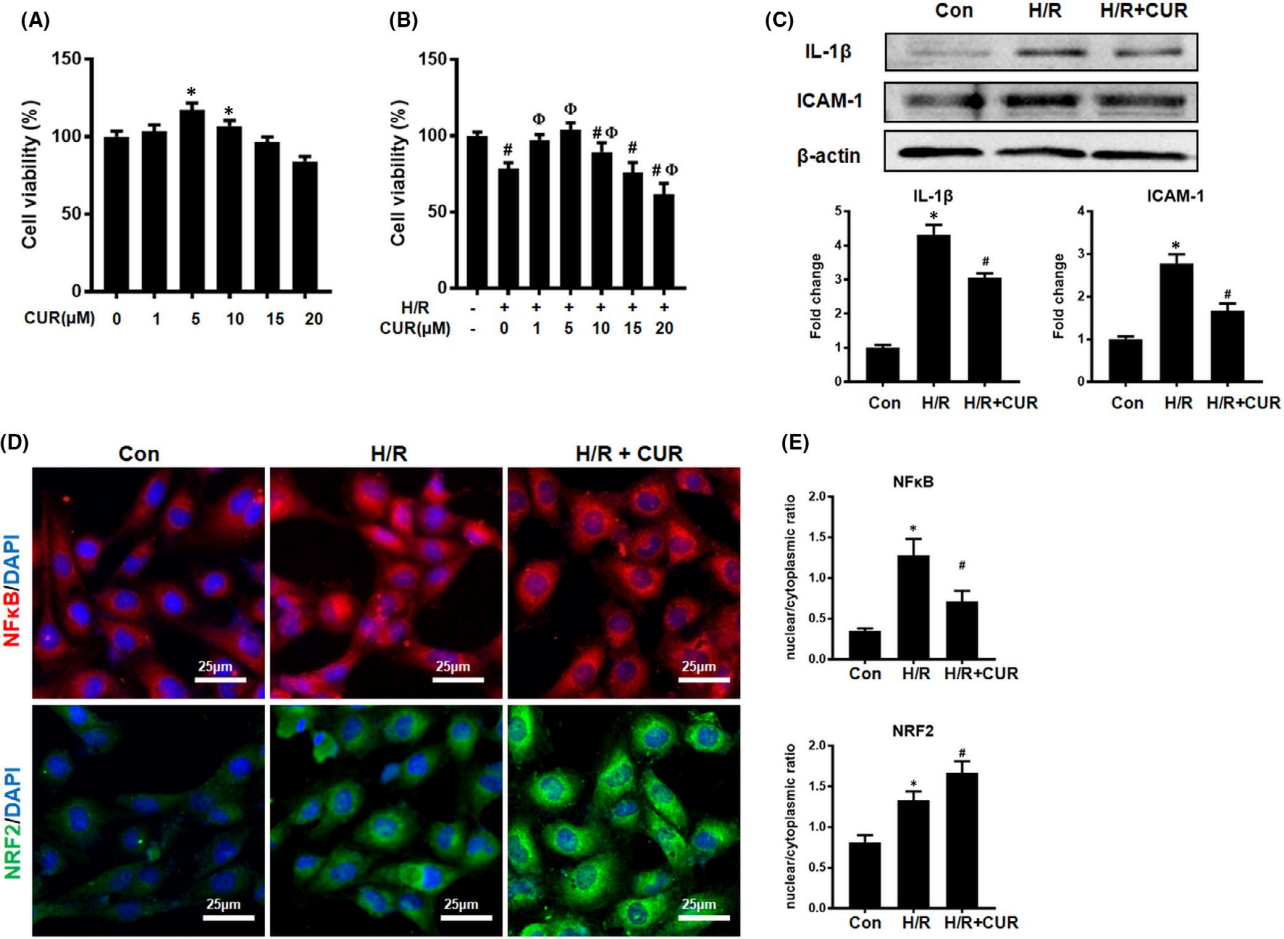
Effect of CUR on cell viability and cytokine production in HK2 cells under H/R conditions. (A and B) Cell viability was determined by CCK‐8 assay. *p < 0.05, vs. 0 μM CUR; ^#^ *p* < 0.05, vs. H/R – and CUR –; ^Ф^p < 0.05, vs. H/R + and 0 μM CUR. (C) Western blot analysis of IL‐1β and ICAM‐1 protein levels. (D) IF staining of NFκB and NRF2 in HK2 cells (scale bar = 25 μm). (E) Quantification of NFκB and NRF2 by IF staining. *p < 0.05, vs. the control group; ^#^ *p *< 0.05 vs. the H/R group (*n* = 3)

As shown in Figure 1A, CUR (at a concentration lower than 10 μM) displayed no obvious cytotoxicity in HK2 cells under normal conditions. In addition, CUR (1–10 μM) treatment improved HK2 cell viability under H/R conditions, and this effect peaked when CUR was applied at a concentration of 5 μM (Figure 1B). Thus, 5 μM CUR was used in the following cell experiments. Moreover, H/R conditions increased the expression of proinflammatory cytokines (ICAM‐1 and IL‐1β) and the nuclear translocation of NFκB in HK2 cells (Figure 1C–E). In contrast, CUR treatment effectively suppressed cytokine expression and NFκB nuclear translocation in HK2 cells under H/R conditions (Figure 1C–E). The increase in NRF2 nuclear translocation under H/R conditions was further enhanced upon treatment with CUR (Figure 1D,E).

### 3.2 CUR restored mitochondrial energy metabolism in HK2 cells under H/R conditions

**FIGURE 2:**
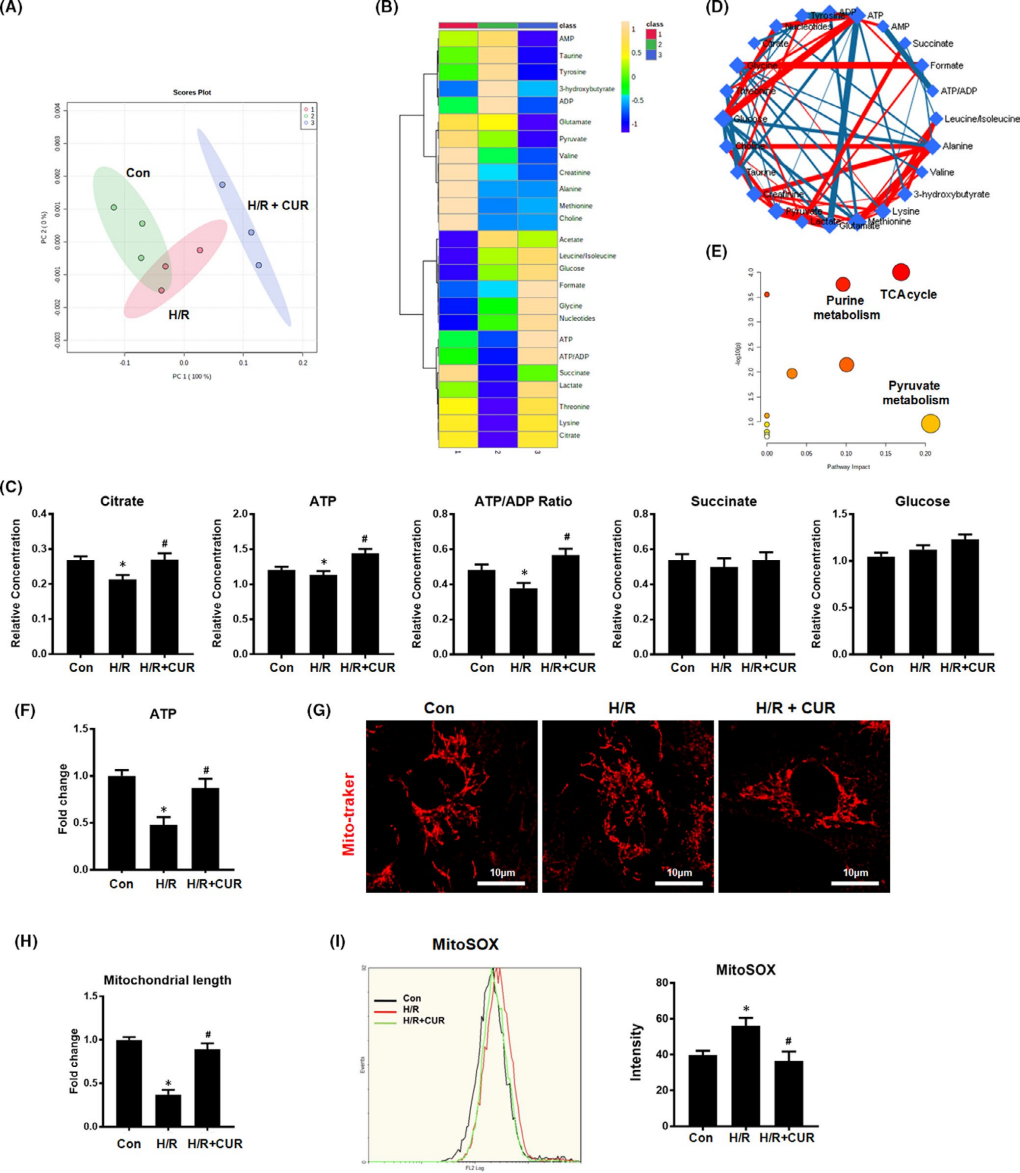
Effect of CUR on mitochondrial metabolism in HK2 cells under H/R conditions. (A and B) PCA score plot and heat map showing different groups based on ^1^H NMR‐based metabolomic results. (C) Relative concentrations of citrate and ATP and the ATP/ADP ratio in HK2 cells. (D and E) Metabolic pathway analysis of differences in metabolite levels between the H/R and H/R + CUR groups. (F) Measurement of ATP levels in HK2 cells. (G and H) Representative micrographs showing MitoTracker staining of HK2 cells (scale bar = 10 µm) and quantification of mitochondrial length. (I) mtROS were measured by flow cytometry with MitoSOX staining. *p < 0.05 *vs*. the control group; ^#^ *p* < 0.05 vs. the H/R group (*n* = 3)

The metabolic profiles of HK2 cells in different groups were analysed by using ^1^H NMR‐based metabolomics, and the identified metabolites were shown in Figure S1. A score plot generated by principal component analysis (PCA) displayed clear separation between the control, H/R and H/R + CUR groups, and a heat map showed that CUR treatment partly abrogated the change in metabolic pattern in the hypoxic HK2 cells compared to HK2 cells subjected to H/R alone (Figure 2A,B). Compared with those in the H/R group, HK2 cells in the H/R + CUR group showed significantly higher citrate and ATP levels and a significantly higher ATP/ADP ratio (Figure 2C). The levels of succinate and glucose in the H/R + CUR group were also slightly increased compared with those in the H/R group, but this difference was nonsignificant (Figure 2C). Further pathway analysis revealed that the TCA cycle and mitochondrial energy metabolism in damaged HK2 cells were enhanced by CUR (Figure 2D,E). To confirm that CUR treatment altered metabolism, changes in mitochondrial function in the HK2 cells were also analysed. Consistently, H/R conditions increased the levels of mtROS and mitochondrial fragmentation and decreased ATP production in HK2 cells in the H/R group compared with the control group, and the mitochondrial lesions in HK2 cells caused by H/R conditions were partly abrogated by CUR treatment (Figure 2F–I).

### 3.3 Pharmacological disruption of mitochondrial homeostasis impaired the anti‐inflammatory capacity of CUR in vitro

**FIGURE 3:**
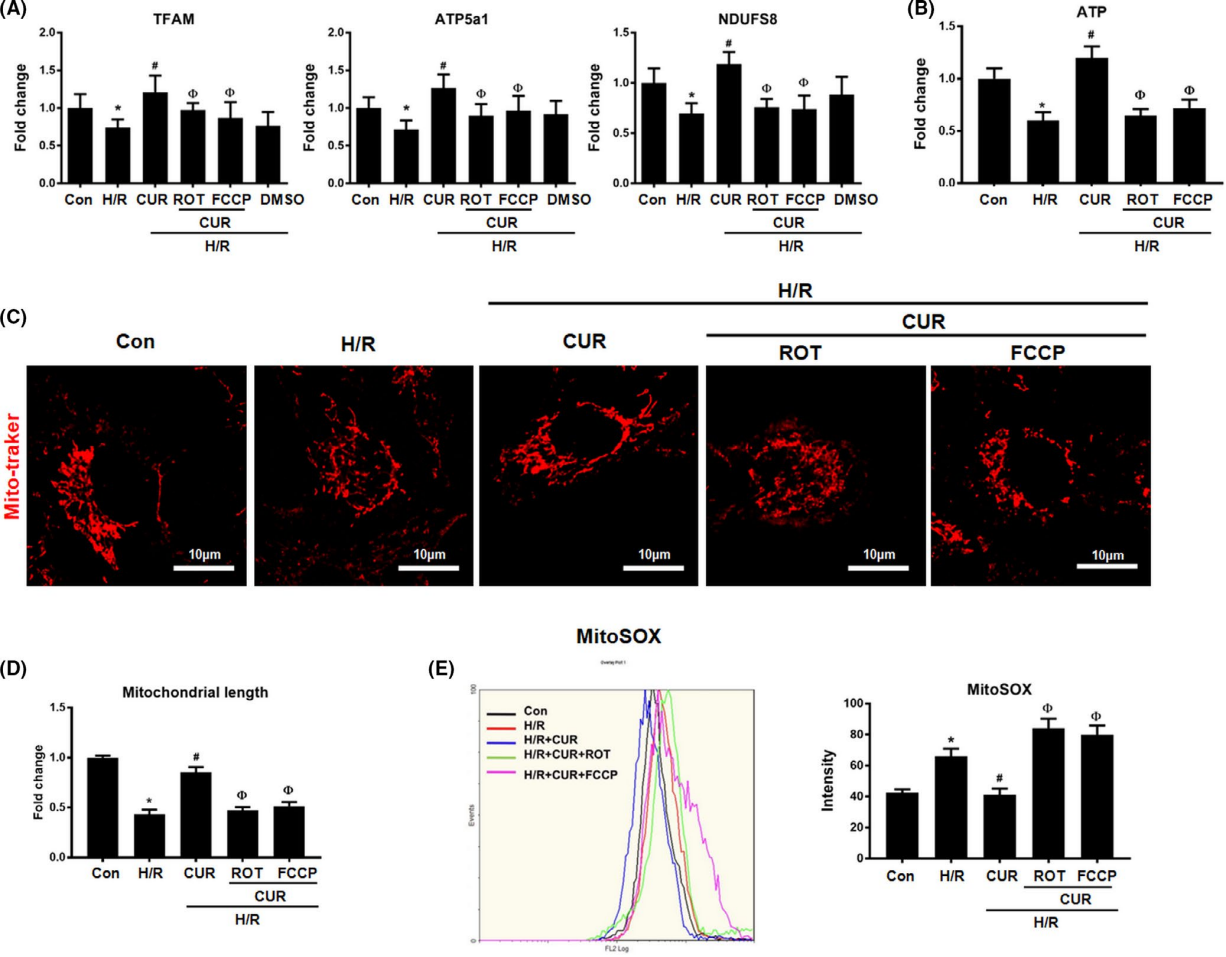
Effect of CUR and ETC inhibitors on mitochondrial function in HK2 cells under H/R conditions. (A) Real‐time PCR analysis of TFAM, ATP5a1 and NDUFS8 mRNA levels. (B) Measurement of intracellular ATP levels in HK2 cells. (C) Representative micrographs showing MitoTracker staining of HK2 cells (scale bar = 10 µm) and (D) quantification of mitochondrial length. (E) Measurement of mtROS levels by flow cytometry with MitoSOX staining. *p < 0.05 vs. the control group; ^#^ *p* < 0.05 vs. the H/R group; ^Ф^ *p* < 0.05 vs. the H/R + CUR group (*n* = 3)

As shown in Figure 3, the decline in mitochondrial function‐related gene (TFAM, ATP5a1 and NDUFS8) expression and ATP production in HK2 cells under H/R conditions was partly restored by CUR treatment, but vehicle (DMSO) treatment had no influence on gene expression or ATP levels (Figure 3A,B). CUR treatment also reduced the levels of mtROS and mitochondrial fragmentation in HK2 cells under H/R conditions (Figure 3C–E). However, the rescue effect of CUR on mitochondrial function in HK2 cells under H/R conditions was largely abolished by cotreatment with ROT (a mitochondrial electron transport inhibitor that disrupts mitochondrial redox balance) or FCCP (a mitochondrial uncoupler that disrupts mitochondrial bioenergetics) (Figure 3A–E).

**FIGURE 4:**
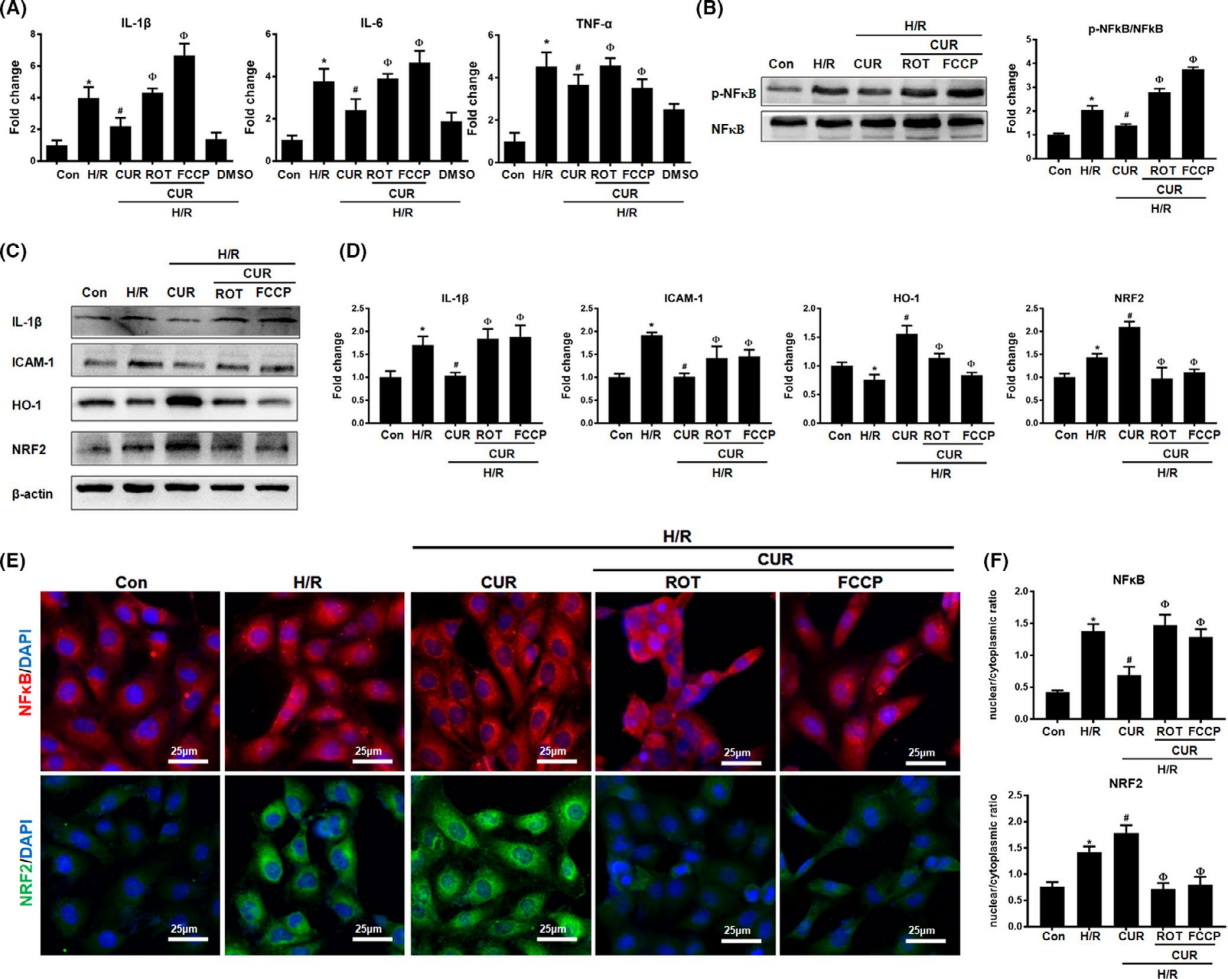
Effect of pharmacological disruption of mitochondrial function on the anti‐inflammatory potential of CUR under H/R conditions. (A) Real‐time PCR analysis of IL‐1β, IL‐6 and TNF‐α mRNA levels in HK2 cells administered different treatments. (B) Western blot analysis and quantification of p‐NFκB levels in HK2 cells. (C‐D) Western blot analysis and quantification of IL‐1β, ICAM‐1, HO‐1 and NRF2 protein levels in hypoxic HK2 cells treated with CUR, CUR +ROT or CUR + FCCP. (E and F) IF staining of NFκB and NRF2 in HK2 cells under H/R conditions administered different treatments (scale bar = 25 μm). *p < 0.05 vs. the control group; ^#^ *p* < 0.05 vs. the H/R group; ^Ф^ *p* < 0.05 vs. the H/R + CUR group (*n* = 3)

We next determined whether the alteration of mitochondrial homeostasis would affect the antioxidant or anti‐inflammatory efficacy of CUR. Compared with those in the control group, CUR treatment reduced levels of the proinflammatory cytokines IL‐1β, IL‐6, ICAM‐1, and TNF‐α and the phosphorylation and nuclear translocation of NFκB in HK2 cells under H/R conditions (Figure 4A–E). Interestingly, the suppressive effects of CUR on cytokine (IL‐1β, IL‐6, TNF‐α and ICAM‐1) production and NFκB phosphorylation in damaged HK2 cells were also markedly abolished upon treatment with ROT or FCCP. Moreover, the upregulation of antioxidant protein (NRF2 and HO‐1) expression and the nuclear translocation of NRF2 induced by CUR in damaged HK2 cells were also eliminated by ROT or FCCP treatment (Figure 4C–E).

### 3.4 Genetic disruption of mitochondrial biogenesis impaired the anti‐inflammatory capacity of CUR in vitro

**FIGURE 5:**
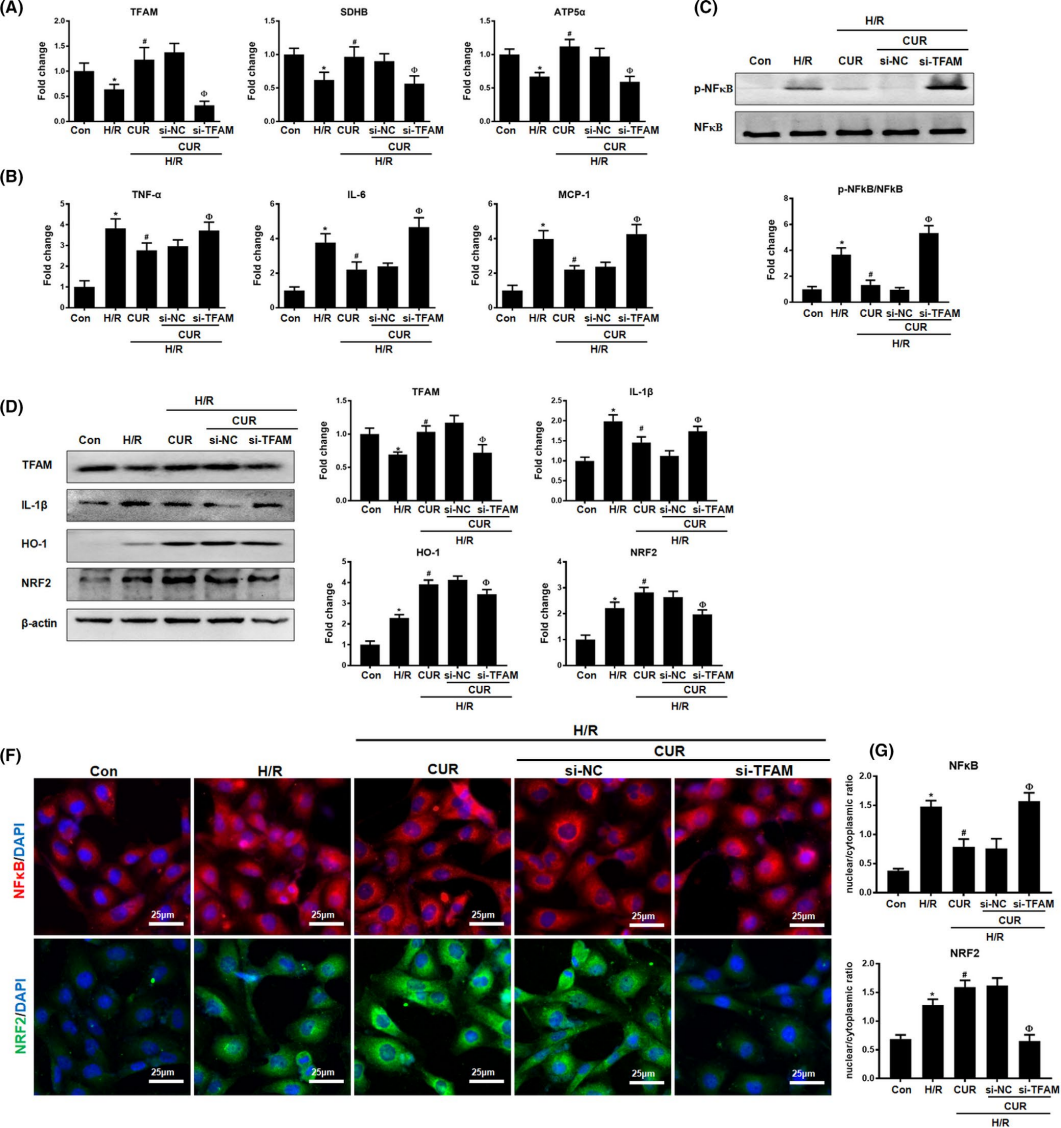
Effect of genetic disruption of mitochondrial biogenesis on the anti‐inflammatory potential of CUR under H/R conditions.(A) Real‐time PCR analysis of TFAM, SDHB and ATP5a1 mRNA levels in HK2 cells treated with CUR, CUR + NC siRNA or CUR + TFAM siRNA. (B) Real‐time PCR analysis of TNF‐α, IL‐6 and MCP‐1 mRNA levels in HK2 cells treated with CUR, CUR + NC siRNA or CUR + TFAM siRNA. (C) Western blot analysis and quantification of p‐NFκB levels. (D and E) Western blot analysis of the TFAM, IL‐1β, HO‐1 and NRF2 proteins in HK2 cells and quantitative analysis of protein expression. (F‐G) IF staining of NFκB and NRF2 in HK2 cells treated with CUR, CUR + NC siRNA or CUR + TFAM siRNA (scale bar = 25 μm). *p < 0.05 vs. the control group; ^#^ *p* < 0.05 vs. the H/R group; ^Ф^ *p* < 0.05 vs. the H/R + CUR group (*n* = 3)

In addition to its disruption via pharmacological tools, mitochondrial biogenesis in HK2 cells was also specifically disrupted by siRNA‐guided TFAM knockdown. Similar to the results reported above, the rescue effects of CUR on mitochondrial function‐related gene (TFAM, SDHB and ATP5a1) expression and the inhibitory effects of CUR on proinflammatory cytokine (TNF‐α, IL‐6 and MCP‐1) expression in damaged HK2 cells were markedly reduced when TFAM was knocked down (Figure 5A,B). TFAM knockdown also impaired the suppressive effects of CUR on the protein expression of p‐NFκB and nuclear translocation of NFκB in HK2 cells under H/R conditions (Figure 5C and F,G). As reported above, the increases in antioxidant protein (HO‐1 and NRF2) levels and NRF2 nuclear translocation induced by CUR in damaged HK2 cells were eliminated when cells were cotreated with TFAM siRNA (Figure 5D–G). There were no significant differences in the mitochondrial function‐related gene, cytokine and p‐NFκB/NRF2 levels the between the CUR + NC siRNA group and the CUR alone group.

### 3.5 Disruption of mitochondrial homeostasis reduced the renoprotective effects of CUR in AKI mice

**FIGURE 6:**
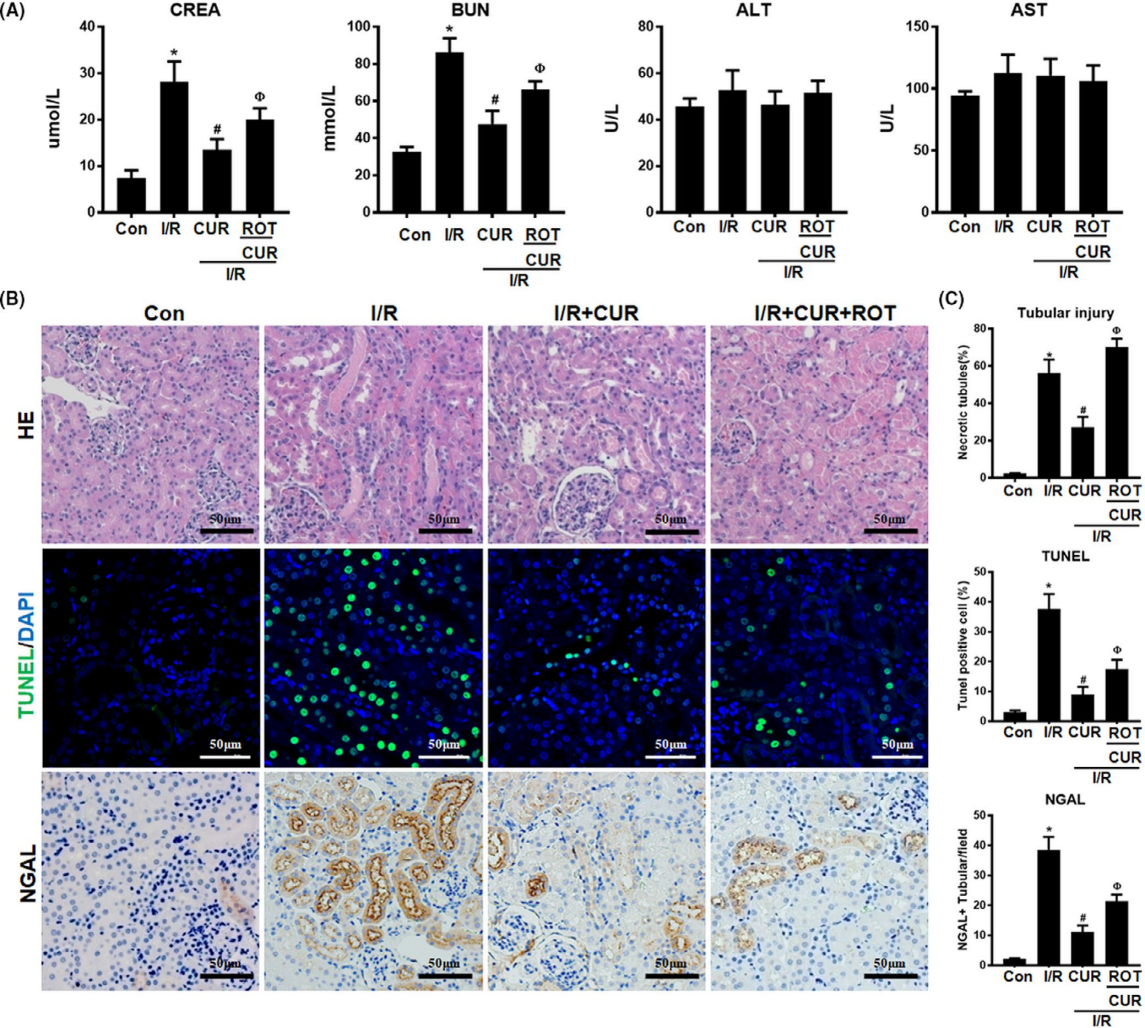
Effect of mitochondrial disruption on the therapeutic potential of CUR in AKI mice. (A) Serum CREA, BUN, ALT and AST concentrations in mice of different groups on day 2 after I/R‐induced AKI. (B) Representative micrographs showing renal haematoxylin and eosin staining (scale bar = 50 µm), TUNEL staining (scale bar = 50 µm), and NGAL staining (scale bar = 50 µm) of the mouse kidneys on day 2 after I/R. (C) Quantitative analysis of necrotic tubules, TUNEL^+^ apoptotic cells and NGAL expression in the kidneys of mice in different groups. *p < 0.05 vs. the control group; ^#^ *p* < 0.05 vs. the I/R group; ^Ф^ *p* < 0.05 vs. the I/R + CUR group (*n* = 6)

Compared with those in the control group, renal function parameters (CREA and BUN levels) were higher in the I/R groups but further decreased in I/R mice treated with CUR. However, I/R mice that received CUR + ROT treatment showed worse renal function than mice in the I/R + CUR group. No significant differences in liver function parameters (ALT and AST) were observed among the four groups (Figure 6A). In addition, compared with the control mice, I/R mice showed increased levels of tubular necrosis, TUNEL^+^ apoptotic cells and kidney NGAL expression, but these renal injuries in the I/R mice were significantly attenuated by CUR treatment (Figure 6B,C). However, the renoprotective effects of CUR in I/R mice were largely weakened when mitochondrial function was disrupted using ROT (Figure 6B,C).

**FIGURE 7:**
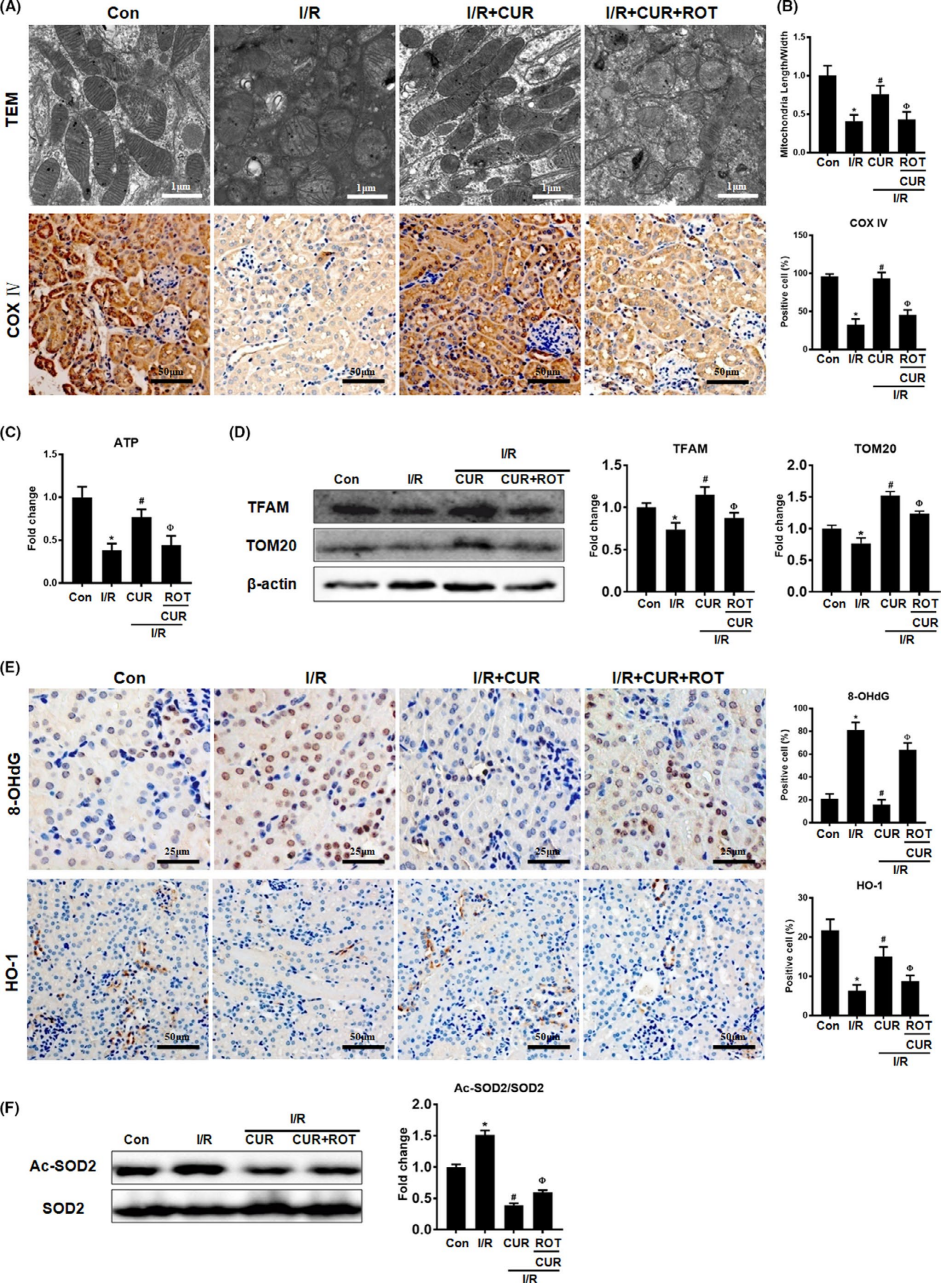
Effect of mitochondrial disruption on the antioxidant potential of CUR in AKI mice. (A) Representative TEM images of mitochondria in the renal tubules of mice (scale bar = 1 µm) and COX IV IHC staining (scale bar = 50 µm) on day 2 after I/R. (B) Quantitative analysis of the mitochondrial length to width ratio detected by TEM and COX IV protein expression. (C) Measurement of ATP levels in the kidneys of mice on day 2 after I/R. (D) Western blot analysis of the TFAM and TOM20 proteins in the kidney. (E) Representative micrographs showing 8‐OHdG IHC staining (scale bar = 25 µm) and HO‐1 IHC staining (scale bar = 50 µm) of the renal cortex and quantitative analysis of 8‐OHdG and HO‐1 levels. (F) Western blot analysis of the Ac‐SOD2/SOD protein ratio in kidneys of mice on day 2 after I/R and quantitative analysis of protein expression. *p < 0.05 vs. the control group; ^#^ *p* < 0.05 vs. the I/R group; ^Ф^ *p* < 0.05 vs. the I/R + CUR group (*n* = 6)

Furthermore, I/R mice displayed obvious renal mitochondrial damage, as indicated by increased mitochondrial swelling, reduced matrix density, and decreased ATP production and mitochondrial biogenesis‐related protein (COX IV, TFAM and TOM20) expression in the kidney compared with those in controls (Figure 7A–D). In contrast, I/R mice that received CUR treatment showed a higher mitochondrial length/width ratio, increased ATP production and increased COX IV/TFAM/TOM20 protein levels than mice in the I/R group (Figure 7A–D). However, the protective effects of CUR on mitochondrial function in the kidneys of I/R mice were also compromised by ROT treatment (Figure 7A–D).

### 3.6 Disruption of mitochondrial homeostasis reduced the antioxidant and anti‐inflammatory effects of CUR in AKI mice

**FIGURE 8:**
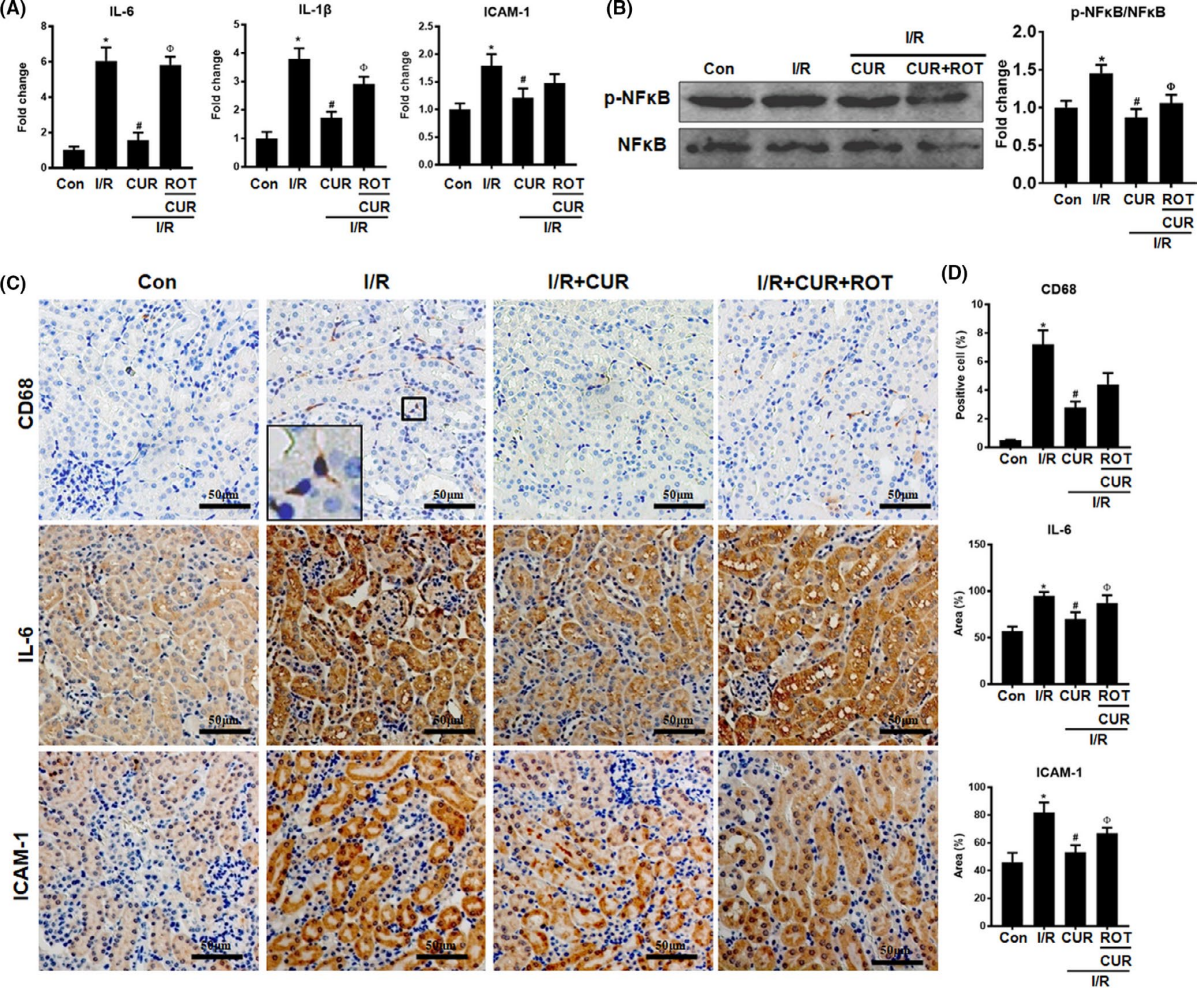
Effect of mitochondrial disruption on the anti‐inflammatory potential of CUR in AKI mice. (A) Real‐time PCR analysis of IL‐6, IL‐1β, and ICAM‐1 mRNA levels in the kidneys of mice on day 2 after I/R. (B) Western blot analysis and quantification of p‐NFκB levels in the kidney on day 2 after I/R. (C) Representative micrographs showing CD68, IL‐6, and ICAM‐1 IHC staining of the mouse kidneys (scale bar = 50 μm). (D) Quantitative analysis of CD68^+^ macrophages and the IL‐6/ICAM‐1 protein expression detected by IHC staining. *p < 0.05 vs. the control group; ^#^ *p* < 0.05 vs. the I/R group; ^Ф^ *p* < 0.05 *vs*. the I/R + CUR group (*n* = 6)

Compared with control mice, I/R mice showed higher levels of an oxidative stress marker (8‐OHdG) and lower levels of an antioxidant protein (HO‐1) in the kidney, but CUR treatment partially reduced the level of 8‐OHdG and increased the level of HO‐1 in the kidneys of I/R mice (Figure 7E). In addition, the Ac‐SOD2/SOD2 protein ratio in the kidney was increased in the I/R group and further reduced by CUR treatment (Figure 7F). However, the antioxidant effects of CUR in the kidneys of I/R mice were limited by ROT treatment (Figure 7E,F). Moreover, I/R mice displayed a hyperinflammatory status, as indicated by increased proinflammatory factor (IL‐6, IL‐1β and ICAM‐1) expression, CD68^+^ macrophage infiltration and p‐NFκB protein levels in the kidney compared with those in the control group (Figure 8A–D). Conversely, CUR treatment partly reduced the levels of proinflammatory factors, CD68^+^ cell number and p‐NFκB protein level in the kidneys of I/R mice. However, the anti‐inflammatory effects of CUR in the kidneys of I/R mice were markedly impaired when mitochondrial function was disrupted using ROT (Figure 8A–D).

## 4 DISCUSSION

Acute kidney injury is a serious disease and very common in critically ill patients,^1^ but no effective therapeutic drugs for AKI are currently available in the clinic.^21^ Therefore, novel treatments that can effectively attenuate AKI are urgently needed. The pathological mechanism of AKI is complicated, and multiple factors, such as oxidative stress, inflammation, apoptosis and mitochondrial damage, have been shown to be involved.^5^ Oxidative stress and inflammation have been proposed as key mediators of the initial phase of I/R‐induced AKI.^22^ Moreover, oxidative stress and inflammation engage in positive feedback via activation of the NFκB pathway, which in turn aggravates renal damage after AKI.^22^ It is well documented that CUR has antioxidant and anti‐inflammatory effects in preclinical models of renal injury. For example, CUR induced NRF2 nuclear translocation and increased antioxidant enzyme (e.g., SOD) levels in 5/6 nephrectomized rats^23^ and inhibited NFκB signalling and the release of inflammatory factors (e.g., TNF‐α and IL‐6) in damaged kidneys.^14^, ^24^ In line with previous reports, we found that CUR effectively improved renal function and reduced renal tubular apoptosis/necrosis in I/R‐induced AKI mice. In addition, CUR reduced oxidative stress markers (mtROS and 8‐OHdG) and increased antioxidant protein levels (NRF2, HO‐1 and SOD2) in vitro and in vivo. CUR treatment also ameliorated inflammatory factor (e.g., IL‐1β and ICAM‐1) expression and NFκB activation in vitro and renal macrophage infiltration/cytokine release in vivo. Altogether, these results suggest that CUR can attenuate ischaemic AKI and that this effect is partly due to its antioxidant/anti‐inflammatory properties.

The kidney is a high‐energy‐demand organ rich in mitochondria, and proper mitochondrial function is necessary to maintain normal renal function.^6^ Mitochondria are not only the main source of energy (ATP) but also participate in many other pathophysiological processes, such as the stress response, apoptosis/death and inflammation.^6^, ^25^ Mitochondrial homeostasis is coordinately regulated by multiple factors, such as mitochondrial biogenesis, redox status and bioenergetics.^25^ Recently, increasing evidence has suggested that mitochondrial damage plays an essential role in initiating inflammation and renal dysfunction after AKI.^6^, ^26^, ^27^ For instance, renal mtDNA damage was found to cause activation of the cGAS‐STING pathway, thereby triggering inflammation, in cisplatin‐induced AKI.^26^ Furthermore, mtROS increases cytokine release and renal injury by disrupting TFAM‐mediated mtDNA maintenance in I/R‐induced AKI.^27^ Thus, mitochondrial dysfunction has been proposed as a promising target for the treatment of AKI. In fact, CUR has shown the potential to preserve mitochondrial functions, such as mitochondrial biogenesis, and the mitochondrial antioxidant machinery in the kidneys of multiple AKI models.^15^, ^16^, ^28^ In line with these reports, the results of our metabolomics analysis show that CUR restored the metabolic profile of damaged TECs, particularly the TCA cycle and ATP production. Further results demonstrated that CUR reduced mitochondrial oxidative stress (mtROS and Ac‐SOD2) and mitochondrial fragmentation and improved mitochondrial biogenesis (TFAM and TOM20) and bioenergetics (ATP synthesis) in damaged TECs and kidneys with AKI. These results suggest that mitochondrial mechanisms also contribute to the renoprotective role of CUR.

Although the anti‐inflammatory and mitochondrial protective effects of CUR have been revealed, the relationship between these effects and consequence of that relationship in AKI remain elusive. Mitochondria can modulate a proinflammatory state via redox‐sensitive inflammatory pathways (e.g., the NFκB pathway) or direct activation of the inflammasome.^29^ Mitochondrial quality and metabolism are tightly regulated by multiple factors, such as mitochondrial redox status, biogenesis and electron transport chain (ETC) activity.^25^ To determine whether mitochondrial dysfunction would affect the anti‐inflammatory potential of CUR in AKI, damaged TECs were cotreated with CUR and pharmacological inhibitors of mitochondrial function. ROT is a mitochondrial ETC complex I inhibitor that induces mitochondrial stress and disrupts mitochondrial redox balance, and FCCP is an oxidative phosphorylation (OXPHOS) uncoupler that blocks ATP synthesis and disrupts mitochondrial bioenergetics.^30^ Interestingly, disruption of mitochondrial homeostasis largely limited the anti‐inflammatory potential of CUR, as evidenced by increased cytokine production and p‐NFκB protein levels in damaged HK2 cells treated with CUR and FCCP or ROT compared to those in the CUR group. In addition, CUR treatment partly restored TFAM expression in damaged HK2 cells and kidneys with I/R. TFAM is a primary mtDNA‐binding protein that regulates mtDNA transcription, thereby controlling mitochondrial biogenesis and OXPHOS.^27^ Thus, we also used a genetic tool (siTFAM) to suppress mitochondrial biogenesis. Similarly, disruption of mitochondrial biogenesis using siTFAM also increased cytokine and p‐NFκB levels in damaged HK2 cells in the presence of CUR. This may have occurred because when the effects of CUR in preserving mitochondria are abolished, innate proinflammatory pathways in damaged mitochondria may be activated by the overproduction of mtROS or release of other damage‐associated molecular patterns (e.g., mtDNA fragments). These results suggest that mitochondrial damage is a driver of the inflammatory process in AKI and that the anti‐inflammatory role of CUR in damaged renal cells is largely dependent on its mitochondrial protective effects.

Moreover, mitochondrial damage can trigger renal cell death by releasing apoptogenic factors or impairing antioxidant mechanisms.^6^ NRF2, a potential target of CUR, is a vital redox stress‐sensitive transcription factor that can induce the expression of many antioxidant and cytoprotective proteins.^13^ CUR was reported to induce NRF2 expression in the kidney in AKI and CKD.^23^, ^31^ Indeed, we found the NRF2‐HO‐1 pathway to be upregulated in damaged TECs or kidneys in response to CUR treatment, but this effect was also largely impaired when mitochondrial homeostasis was strongly disrupted by pharmacological means or genetic manipulation. These findings indicate potential crosstalk between the NRF2 signalling pathway and mitochondrial homeostasis, since the activity of Nrf2 was reported to be regulated by mitochondrial redox status.^32^, ^33^ In addition, mitochondria are the major source of intracellular ROS (>90%), and SOD2 is a key mitochondrial antioxidant protein that converts superoxide (O_2_^−^) into hydrogen peroxide. The activity of the SOD2 protein is also affected by mitochondrial function, and hyperacetylation of SOD2 leads to elevated levels of O_2_^−^ in mitochondria.^34^ Interestingly, Ac‐SOD2 levels in the kidneys of AKI mice were decreased by CUR, but this effect was markedly eliminated upon mitochondrial disruption. However, exploration of the underlying mechanism in future studies is required. Taken together, our results suggest that the therapeutic role of CUR in AKI is primarily dependent on mitochondrial mechanisms (e.g.,` redox balance, biogenesis and bioenergetics) and that mitochondria are indispensable for maintaining the antioxidant and anti‐inflammatory effects of CUR in AKI.

## 5 CONCLUSION

**FIGURE 9:**
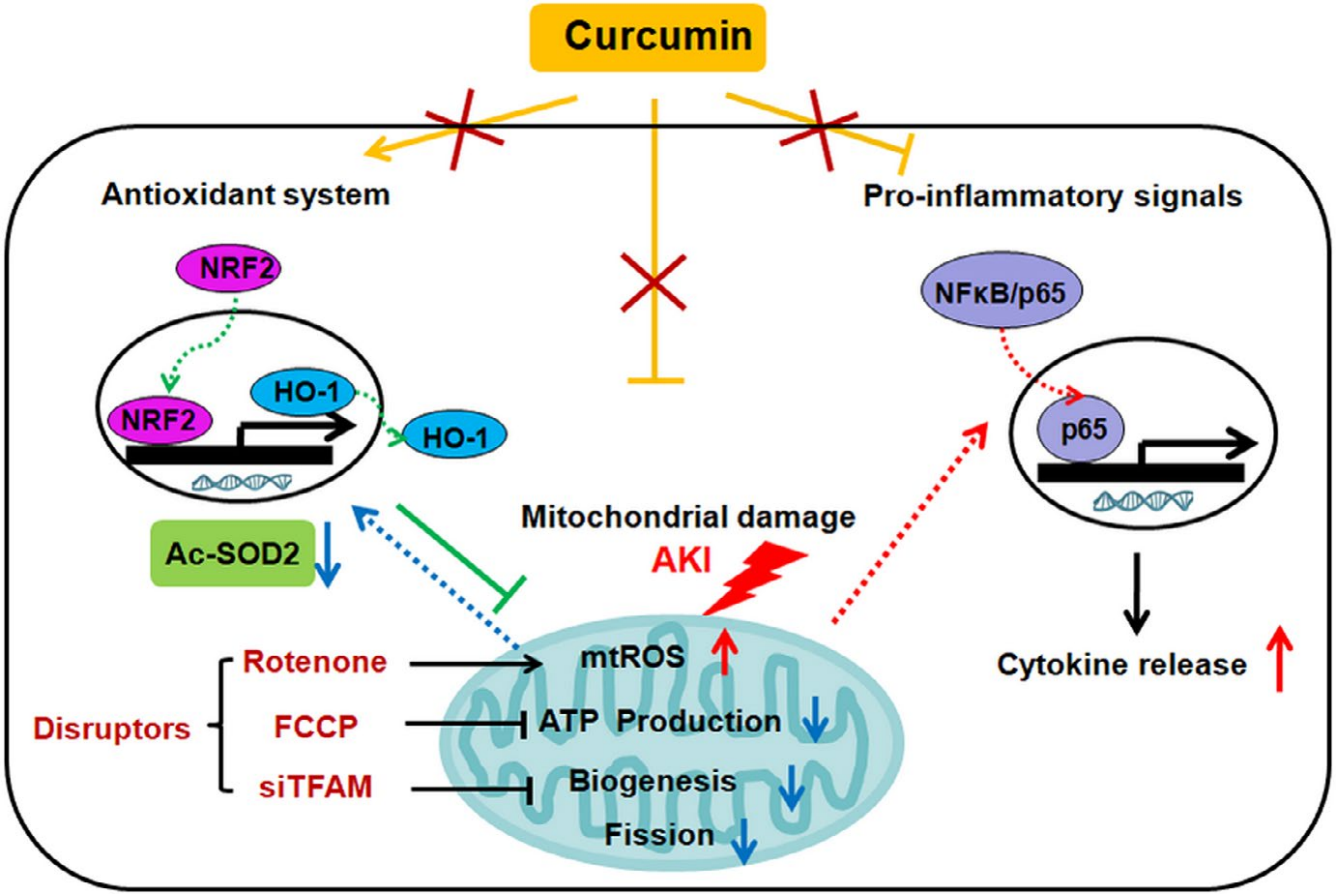
The proposed findings of this study. CUR exerts multiple benefits in AKI, including its antioxidant (induction of NRF2/HO‐1 pathway and SOD2 deacetylation), anti‐inflammatory (inhibition of NFκB activation and cytokine release) and mitochondrial protective (reduction in mtROS and mitochondrial fragmentation, increase in biogenesis and ATP synthesis) effects. However, mitochondrial disruption by ETC inhibitors or siTFAM markedly reduced the antioxidant and anti‐inflammatory potential of CUR. This study indicates that mitochondrial damage is a driver of AKI and that the therapeutic role of CUR in AKI is primarily dependent on mitochondrial pathways

In summary, our results demonstrate that CUR treatment attenuated inflammation, oxidative stress and mitochondrial damage in kidneys subjected to I/R‐induced AKI. Furthermore, CUR restored mitochondrial redox balance, biogenesis and bioenergetics in damaged TECs and kidneys, while disruption of mitochondrial function by pharmacological means or genetic manipulation markedly decreased the antioxidant and anti‐inflammatory effects of CUR (Figure 9). This study suggests that CUR ameliorates renal injury primarily by preserving mitochondrial homeostasis and that CUR may be an alternative therapy for other diseases characterized by mitochondrial dysfunction.

## CONFLICT OF INTEREST

The authors declare no conflicts of interest.

## AUTHOR CONTRIBUTION

**Ling LI:** Data curation (equal); Formal analysis (equal); Methodology (equal); Writing‐original draft (lead). **Shuyun Liu:** Data curation (equal); Formal analysis (equal); Investigation (equal); Methodology (equal). **Yijie Zhou:** Methodology (equal). **Meng Zhao:** Methodology (equal). **Yizhuo Wang:** Methodology (equal). **Chengshi Wang:** Data curation (equal). **Peng Lou:** Methodology (equal). **Rongshuang Huang:** Data curation (equal). **Liang Ma:** Methodology (equal). **Yanrong Lu:** Writing‐original draft (equal). **Ping Fu:** Writing‐review & editing (equal). **Jingping Liu:** Data curation (equal); Investigation (equal); Methodology (lead); Project administration (lead); Resources (lead); Supervision (equal); Writing‐review & editing (lead).

## Supporting information

Supplementary Material

## Data Availability

The data that support the findings of this study are available from the corresponding author upon reasonable request.
