# Supplementary material for: Indispensable role of mitochondria in maintaining the therapeutic potential of curcumin in acute kidney injury
Source: J Cell Mol Med. 2021 Sep 16;25(20):9863–77. doi: 10.1111/jcmm.16934 (PMC8505835; doi:10.1111/jcmm.16934)

**Supporting Information**

Indispensable role of mitochondria in maintaining the therapeutic potential of curcumin in acute kidney injury

Ling Li^2^, Shuyun Liu^1^, Yijie Zhou^1^, Meng Zhao^1^, Yizhuo Wang^1^, Chengshi Wang^1^, Peng Lou^1^, Rongshuang Huang^2^, Liang Ma^2^, Yanrong Lu^1^, Ping Fu^2^, Jingping Liu^1*^

^1^ Key Laboratory of Transplant Engineering and Immunology, National Clinical Research Center for Geriatrics, Frontiers Science Center for Disease-related Molecular Network, West China Hospital of Sichuan University, Chengdu, China

^2^ Division of Nephrology and National Clinical Research Center for Geriatrics, Kidney Research Institute, West China Hospital of Sichuan University, Chengdu China

^*^Corresponding author: Jingping Liu, Key Laboratory of Transplant Engineering and Immunology, West China Hospital, Sichuan University, No. 1 Keyuan 4th Road, Gaopeng Ave, Chengdu 610041, China. Tel.: +86-28-85164029; Fax: +86-28-85164030; E-mail: liujingping@scu.edu.cn.

**Supplemental Results**

**
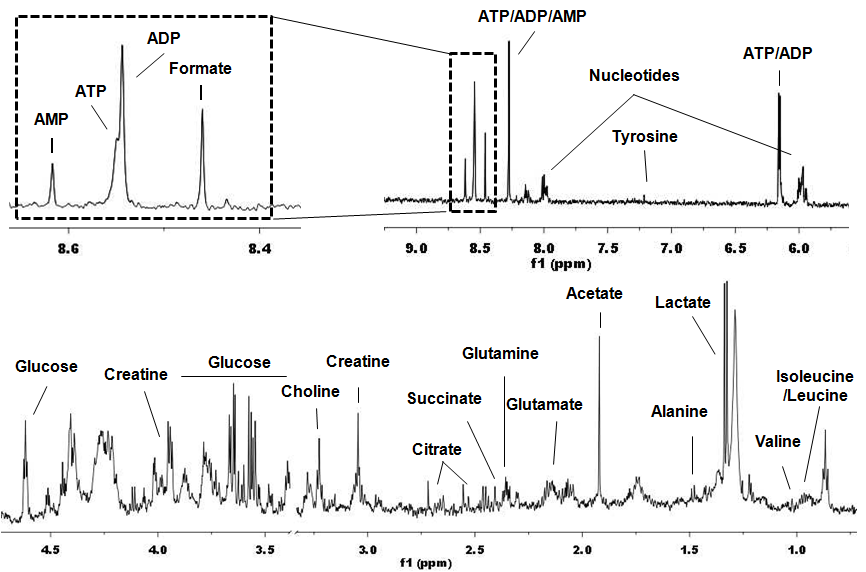
**

**Fig. S1** Representative 600-MHz ^1^H NMR spectra of aqueous cell extracts from normal cultured HK2 cells. The identified metabolites are marked.

**Table S1**. Real-time PCR primers used in the study

| Gene | Sequence 5'-3' | Species |
| --- | --- | --- |
| TFAM-F | AGCTCAGAACCCAGATGCAA | Human |
| TFAM-R | CCGCCCTATAAGCATCTTGA |  |
| ATP5a1-F | AGAGGACAGGAGCCATTGTG |  |
| ATP5a1-R | TCAGACCAACTCGCCTACG |  |
| NDUFS8-F | CATCTACTGCGGCTTCTGC |  |
| NDUFS8-R | GGGCGTCACCGATACAAGT |  |
| SDHB-F | ATCTTGTTCCCGATTTGAGC |  |
| SDHB-R | TAGAGCCCGTCCAGTTTCTC |  |
| IL-1β-F | TGGCAGAAAGGGAACAGAAA |  |
| IL-1β-R | CTGGCTGATGGACAGGAGAT |  |
| IL-6-F | GAAAGCAGCAAAGAGGCA |  |
| IL-6-R | CACCAAGTTGAGGGAATGA |  |
| TNF-ɑ-F | TGCTGCACTTTGGAGTGATCG |  |
| TNF-ɑ-F | TGTCACTCGGGGTTCGAGAAG |  |
| MCP-1-F | GATCTCAGTGCAGAGGCTCG |  |
| MCP-1-R | TGCTTGTCCAGGTGGTCCAT |  |
| β-actin-F | CCACGAAACTACCTTCAACTCC |  |
| β-actin-F | GTGATCTCCTTCTGCATCCTGT |  |
| IL-6-F | AAATGATGGATGCTACCAAACT | Mouse |
| IL6-R | CTCTGGCTTTGTCTTTCTTGTT |  |
| IL-1β-F | GTCCTGTGTAATGAAAGACGGC |  |
| IL-1β-R | CTGCTTGTGAGGTGCTGATGTA |  |
| ICAM-1-F | ACCCAACTGGAAGCTGTTTG |  |
| ICAM-1-R | CACACTCTCCGGAAACGAAT |  |
| β-actin-F | CGTGCGTGACATCAAAGAGAA |  |
| β-actin-F | AACCGCTCGTTGCCAATAGT |  |

The original western blotting images used in the study


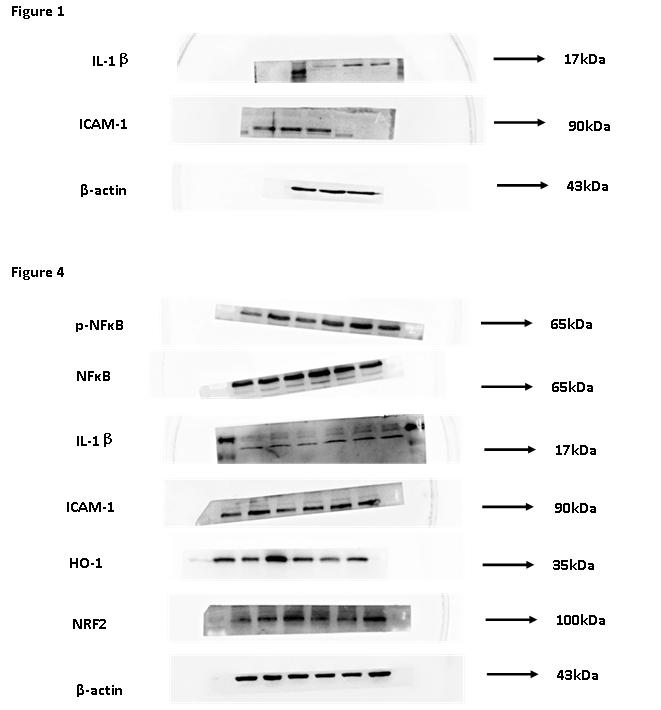

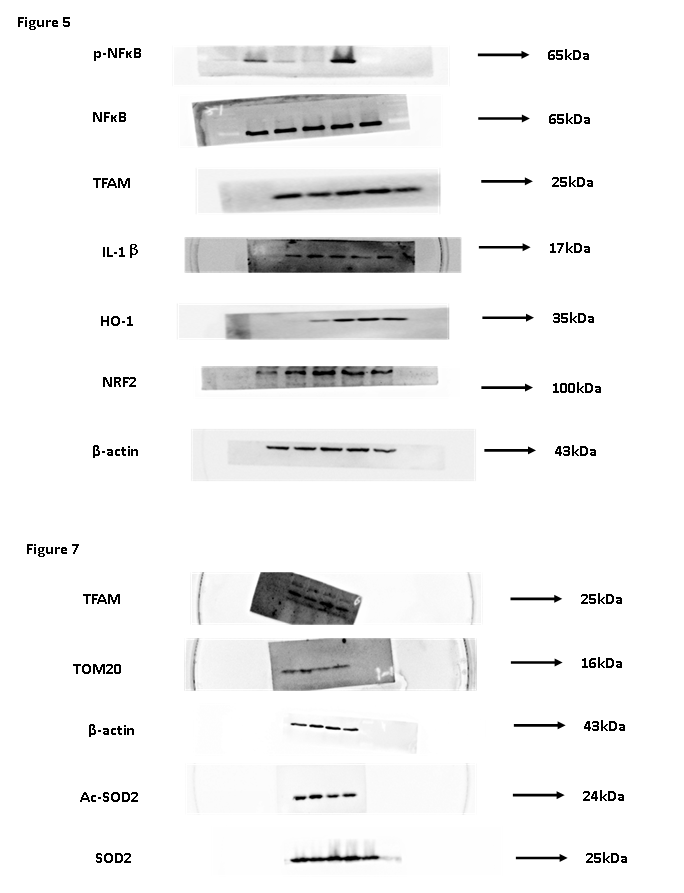

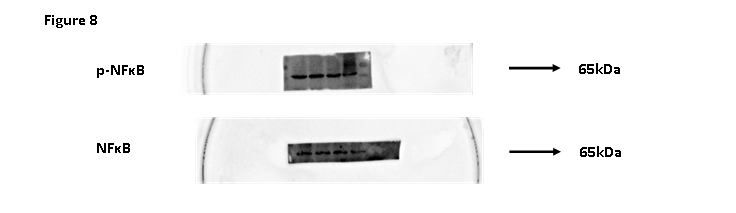

Supplement: Supplementary file 1 — Supplementary Material [file JCMM-25-9863-s001.docx]
